# Mechanochemical amorphization of chitin: impact of apparatus material on performance and contamination

**DOI:** 10.3762/bjoc.15.119

**Published:** 2019-06-05

**Authors:** Thomas Di Nardo, Audrey Moores

**Affiliations:** 1 Centre in Green Chemistry and Catalysis, Department of Chemistry, McGill University, 801 Sherbrooke St. West, Montreal, QC, H3A 0B8, Canada

**Keywords:** amorphization, biomass, chitin, hardness, mechanochemistry, metal contamination

## Abstract

Herein, we present a study of the impact of the jar and ball medium on the performance in the mechanochemical amorphization of chitin. We measured the crystallinity index of chitin after milling it in a vibration mill in an apparatus made of copper, aluminum, brass, tungsten carbide, zirconia, stainless steel, polytetrafluoroethylene (PTFE), or poly(methyl methacrylate) (PMMA). These materials offer a range of Vickers hardness values and the impact of these parameters is discussed. The role of the size and mass of the balls is also studied in the case of stainless steel. This study also highlights one of the major challenges during milling, which is contamination of the studied samples.

## Introduction

The last decade has seen a tremendous development in the field of mechanochemistry, which was notably covered in the *New York Times* in 2016 [1]. Yet, mechanochemistry is hardly a novel concept, since it is likely among the most ancient methods for human beings to practice chemistry. Indeed the ability to produce fire is considered a crucial landmark for human development, and archeological evidences indicate that anthropogenic fire production relied on wood-on-wood friction or on percussion or friction of a siliceous rock against pyrite [2–3]. Mechanochemistry comes with intrinsic advantages associated with the lack of solvent use during reaction, thus largely reducing the generation of waste [4] even in active pharmaceutical ingredient synthesis [5]. Yet mechanochemistry also allows the production of novel materials, which are distinct from what is enabled via solvothermal methods [6–8]. Finally, it also opens novel opportunities to react and functionalize solvent recalcitrant materials [9–13], including biomass-based materials [14–25]. Although the technique has been demonstrated to be useful, there are still many questions yet to be answered on the effects of reaction conditions on performance in mechanochemical systems. A number of groups have looked into better understanding the role of temperature, mixing frequency, pressure, reactor medium and atmosphere to relative degrees depending on equipment [5,26–34]. In particular, the Mack group has used an elegant strategy to evaluate the energy delivery for a Diels–Alder reaction performed under mechanochemical conditions and was able to correlate reactivity, or lack thereof, with the specific reaction kinetic parameters [33]. There is, however, an interest in investigating these aspects further. In particular, it would be interesting to track the role of parameters such as ball size and mass on the progress of the reaction. In mechanochemistry, it is accepted that chemical reactions are induced by the delivery of energy, by collision. The impact force is thus of importance and depends on the weight of the balls and [26] the surface of impact [28]. Also, in the context of the milling of crystalline and/or fairly hard materials, the nature of the milling media composing the balls and jars, and their hardness parameter, should have an impact [33], an effect that needs better documentation.

Additionally, mechanochemical treatment may cause materials to be contaminated by the apparatus material through wear from friction and impact. This wear would incidentally leave residual amounts of material from the jars and balls in the reagents and products as a contamination. Milling media can have a catalytic effect due to leaching or surface adsorption [7,35–37]. Contamination has been noted in alloying [26,38] as well as amorphization of hard materials [39], and could cause doping effects in coordination polymers [40–41]. Yet a systematic study of quantitative contamination as a function of the medium has not been reported.

Mechanochemistry is a promising strategy towards the activation, functionalization and deconstruction of biomass. Mechanochemical activation can expedite polysaccharide hydrolytic cleavage [20–23]. Milling can also facilitate chitin depolymerization [24] and when used with base simultaneously deacetylate and depolymerize chitin [42]. One of the key processes in the context of biomass upgrading is amorphization. Indeed, both chitin and cellulose are extracted as highly crystalline material and their mechanochemical amorphization has been explored as a means to favor subsequent depolymerization [16], or functionalization [18]. For functionalization of these materials, it has been shown that amorphization as a pretreatment can be utilized to expose more functional groups to the surface, increasing the rate of reaction. Amorphization can also be used for better materials packing increasing hardness after sintering [17].

This study is important as the choice of milling media may be conditioned by the need to select, for instance, transparent jars to perform in situ measurements [6,43–47]. In these cases, better understanding of milling ball choice would allow one to control energy input in the system as well as minimize jar damage and contamination. Creative setups have also been developed where the measurement window lies outside of the milling chamber where sample can pass in and out for continuous monitoring [48]. This allows for varying milling media with the benefit of in situ measurements.

For some processes like reactive aging with enzymes [22], polytetrafluoroethylene (PTFE) jars were preferred over stainless steel ones, since the latter caused the reactive mixture to adhere to the jars, which was not the case of the former. In another example, for the metal-free transfer hydrogenation of carbonyl compounds, PTFE was used instead of steel to eliminate the possibility of catalysis from the milling media [9]. In these cases, milling is used as a mixing method, whereby the reactions did not require large activation energies to be driven forward [33]. Soft and hard milling can be considered from a multitude of parameters where ball to powder ratio (BPR) is used as a way to “concentrate” or “dilute” kinetic energy from the milling media by reducing or increasing the milled powder ratio [26]. Furthermore, the size of the jar, the size, mass [28] and number of balls [29,49–50], as well as jar filling [26,30] will affect kinetic energy transfer. Although some comparisons on milling media hardness have been considered and showed an effect on the yield [29–31], to the best of our knowledge, there are no larger studies comparing several materials, especially with consideration to biomass amorphization and contamination.

Amorphization is a common process whereby the crystallinity of a material is reduced by mechanical forces [17,24,50–51] or otherwise [52] by deforming particles and breaking lattice imperfections [50]. For biomass processing, amorphization has been used in several studies [24,53–54], either as a pretreatment [21] prior to deacetylation [18,55], enzymatic [22,56–57] or acidic depolymerization [23–24] and simultaneously during processing [42], yielding oligomers and monomers. Differentiating these regions of impact would be of great value for biomass processing. It is therefore important to understand precisely how to master media selection: finding the right combination of balls, size and apparatus materials can help to optimize the energy input while allowing the desired loss of crystallinity for hard milling or maintaining crystallinity for soft milling techniques.

In order to better understand the role of the milling medium on polymer reactivity, we launched a systematic study of the effects of a number of medium parameters onto a model reaction, namely the amorphization of chitin where interchain stabilization is greater than 250 kJ/mol based on density functional theory (DFT) calculation [58]. We explored the role of the material of the jar and the ball, ball size and mass, while concentration on amorphization performance, measured by powder X-ray diffraction (PXRD). We selected for this study reaction jars made of copper, aluminum, brass, tungsten carbide, zirconia, stainless steel, PTFE, or poly(methyl methacrylate) (PMMA) paired with one ball of the same composition with either a diameter of 9.5 mm or mass of approximately 2 g. We could determine that Vickers hardness is a key parameter determining the ability to perform amorphization of biomass materials especially when considering efficient kinetic energy transfer [59]. Finally, the effect of materials contamination on mills samples was tested by inductively coupled plasma optical emission spectrometry (ICP–OES) and X-ray photoelectron spectroscopy.

## Results and Discussion

### Study of chitin amorphization as a function of medium hardness

**Figure 1:**
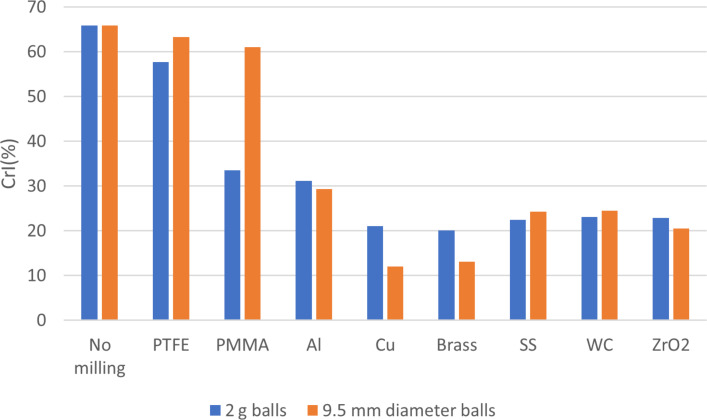
CrI% for chitin samples, before and after milling in a jar with one ball made of SS, ZrO_2_, Cu, Al, brass, WC, PMMA, PTFE. One series has ball mass = 2 g (blue) and one ball diameter = 9.5 mm (in orange). Experimental conditions: 200 mg of chitin, milling time: 30 min, milling frequency 29.5 Hz.

In this study, we probed the effect of milling parameters on the performance of chitin amorphization. As a first experiment, we wanted to screen the effect of the milling media. In a typical experiment, a jar was filled with 200 mg of shrimp PG chitin, fitted with one ball of the same material as the jar and milled in a vibrational mill for 30 min. Eight milling media were tested: stainless steel (SS), zirconia (ZrO_2_), copper (Cu), aluminum (Al), brass, tungsten carbide (WC), poly(methyl methacrylate) (PMMA), and polytetrafluoroethylene (PTFE). These media have distinct density; we needed to normalize the resulting experiments. We expected two ball parameters to play a role in the process: the contact surface area of the ball with the jar (impact density), which depends on the ball size, and the mass of the ball, which impacts the kinetic energy delivered during milling. We thus ran two series of tests with balls of different materials (mentioned above), one where ball mass was chosen to be as close as possible to 2 g (2.16 +/− 0.22 g, see Table S1, Supporting Information File 1 for exact values), while its diameter varied based on density, and one with balls of 9.5 mm in diameter which vary in mass also based on density. Both fixed parameters were chosen based on the availability of such milling balls in our inventories or from suppliers, and based on physical constraints. The lowest density material in our study, PMMA, has a 2 g ball diameter of 16 mm, which just fits the inner diameter of the jar, i.e. 19 mm. Amorphization progress was followed by PXRD. Characterization of the crystallinity index (CrI) of chitin can be determined by comparing the area of the crystalline region to the global area in the PXRD spectrum (Figure S2, Supporting Information File 1) [60]. Untreated chitin powder CrI was measured to be 65.8%. CrI of samples after the milling reaction were plotted as a function of the medium for each of the two series (Figure 1). A clear trend was observed with the effect of material hardness on amorphization. PTFE afforded poor amorphization, whether we look at 2 g balls or 9.5 mm ones. Al, Cu, brass, SS, WC and ZrO_2_ were all performing well to reduce crystallinity by at least a half and give a range of values between 10 and 30%. PMMA gave an intermediate result, whereby 9.5 mm (0.5349 g) gave poor amorphization (61%) while the 2 g equivalent (2.4588 g) afforded a good one (33.5%).

**Figure 2:**
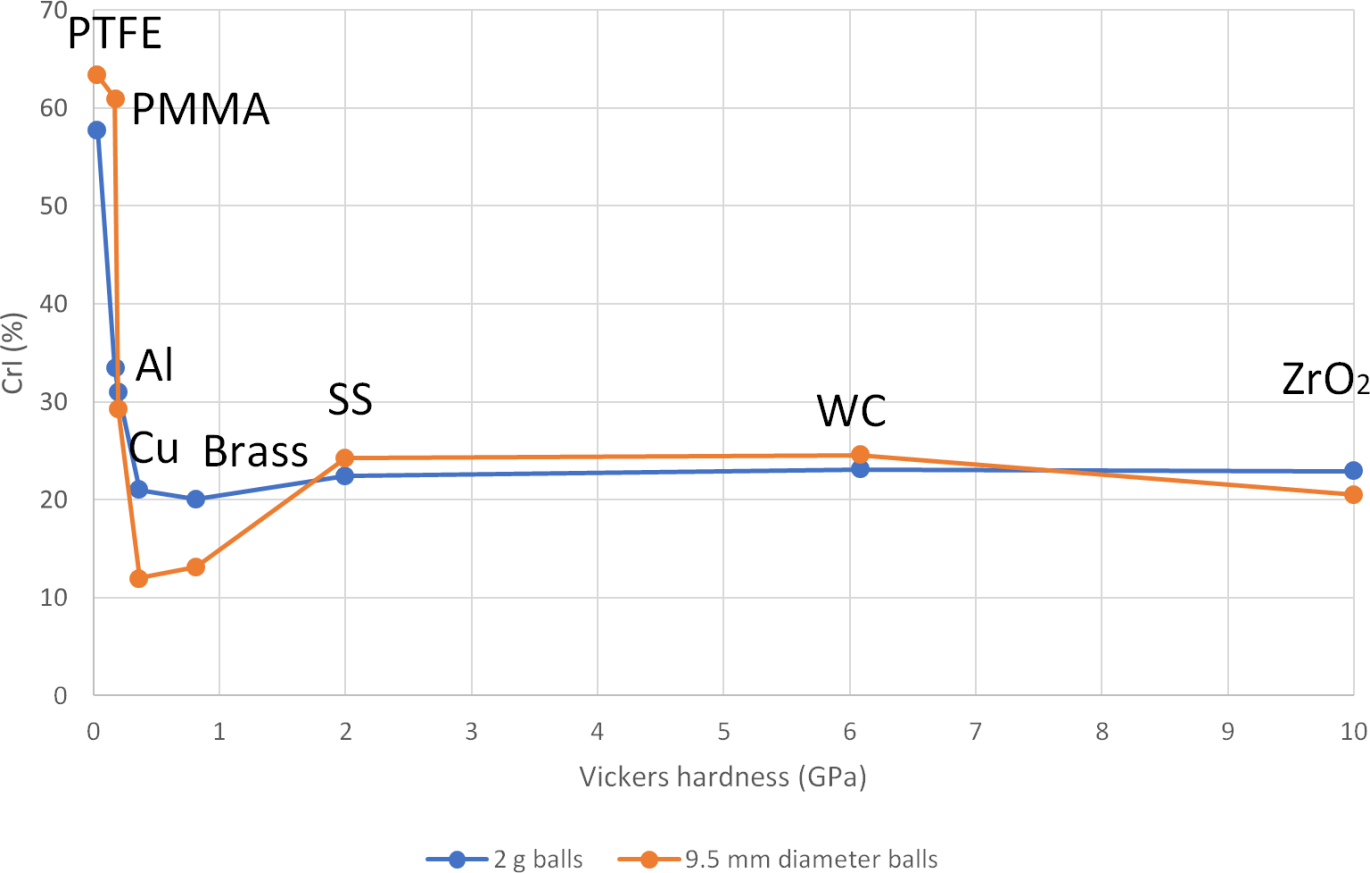
Value of CrI after milling as a function of the Vickers hardness values of the media (Jar and ball) used in this study. Two series are compared: One where the balls are 2 g, and one where they are 9.5 mm in diameter. Experimental conditions: 200 mg of chitin, milling time: 30 min, milling frequency 29.5 Hz.

The different materials tested in this experiment feature very distinct hardness values. We retrieved Vickers hardness values from material specification sheets to correlate amorphization performance with them (Figure 1). With the 2 g ball series, a clear trend can be seen. A crossover point exists where all materials of Vickers hardness larger than the one of chitin itself, which was estimated to be 25–80 kgf/mm^2^ (245–784 MPa) from chitin insect cuticle [61], performed very well for the amorphization, while materials of lower hardness hardly affected the crystallinity of the substrate. Interestingly, when comparing the balls by size, Cu and brass gave really low values of crystallinity, despite a middle range hardness. This indicates that the comparison by mass is more relevant. Cu and brass are fairly dense materials, which explain their good performance, particularly for the 9.5 mm series where their mass would comparatively be more significant (Figure 2).

**Figure 3:**
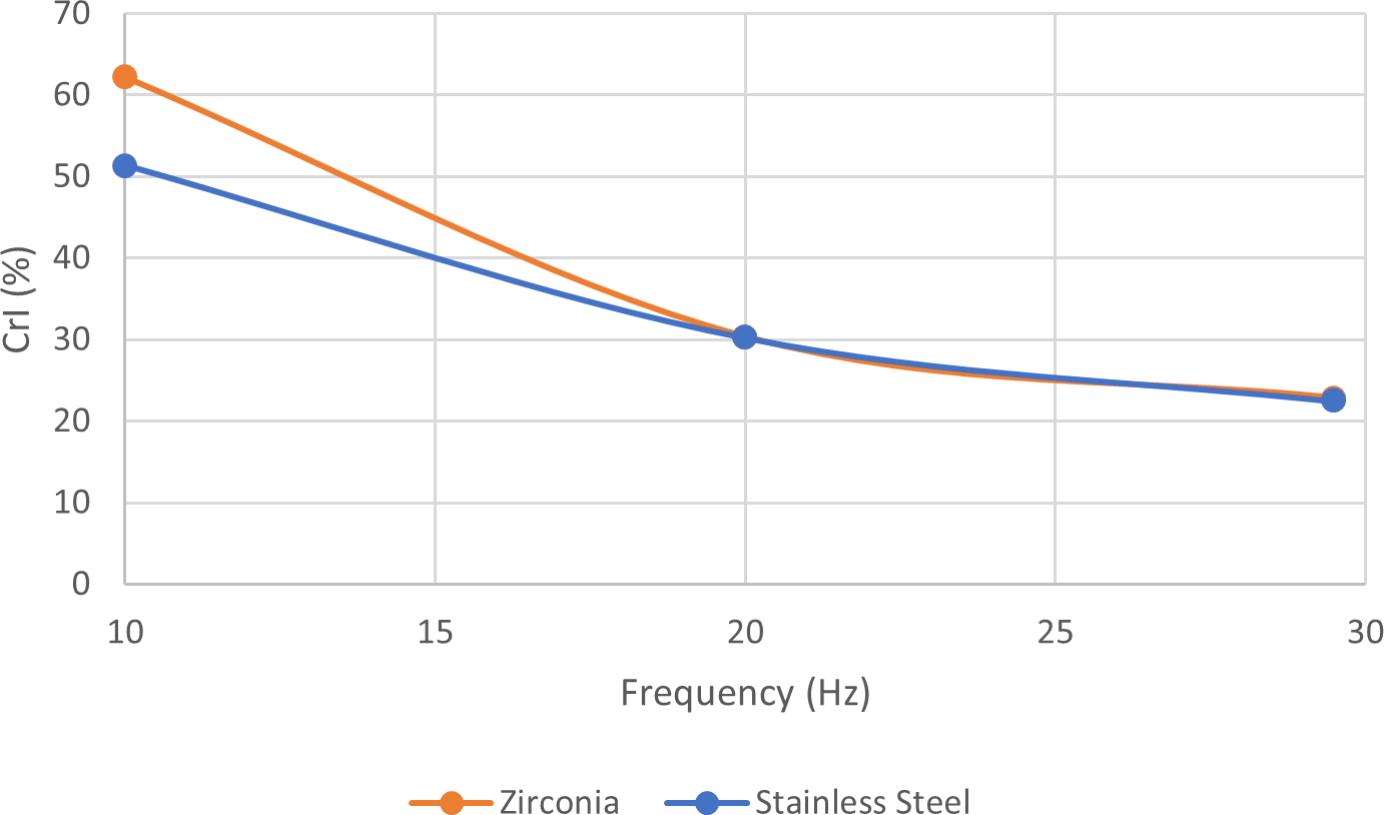
Crystallinity index (%) after milling as a function of frequency. ZrO_2_ and SS jars are compared, both with 1 2 g ball of the same material. Experimental conditions: 200 mg of chitin, milling time: 30 min, milling frequencies varied from 10, to 20 and 29.5 Hz.

It was interesting to note that while the Vickers hardness of SS, WC and ZrO_2_ are vastly different, these three materials seem to behave similarly as jar and ball mechanochemical media for the studied reaction. We were curious to investigate if varying the frequency of milling could reveal an effect of these materials density. We thus selected SS and ZrO_2_ media and varied the frequency from 10, to 20 and finally 29.5 Hz (Figure 3). When milling at 29.5 Hz, with a ≈2 g ball in ZrO_2_ and SS (Table S1, Supporting Information File 1 for exact mass values), little difference was observed in the CrI of chitin after milling, achieving values of 22.9 and 22.4, respectively. Decreasing the frequency decreases the kinetic energy delivered from the ball providing greater differentiation between ball mass and density in terms of amorphization efficiency. Decreasing the frequency to 20 Hz maintained overall higher CrI of 30.4 and 30.2 for ZrO_2_ and SS, respectively. At 10 Hz, however, we clearly observed a difference in the resulting CrI, reaching 62.3 and 51.4, for ZrO_2_ and SS, respectively. At this frequency, the lower density material, ZrO_2_ (6.0 g/cm^3^) afforded less amorphization than the denser one, SS (7.7 g/cm^3^). At this frequency the impact force seems to play a more important role in amorphization efficacy.

**Figure 4:**
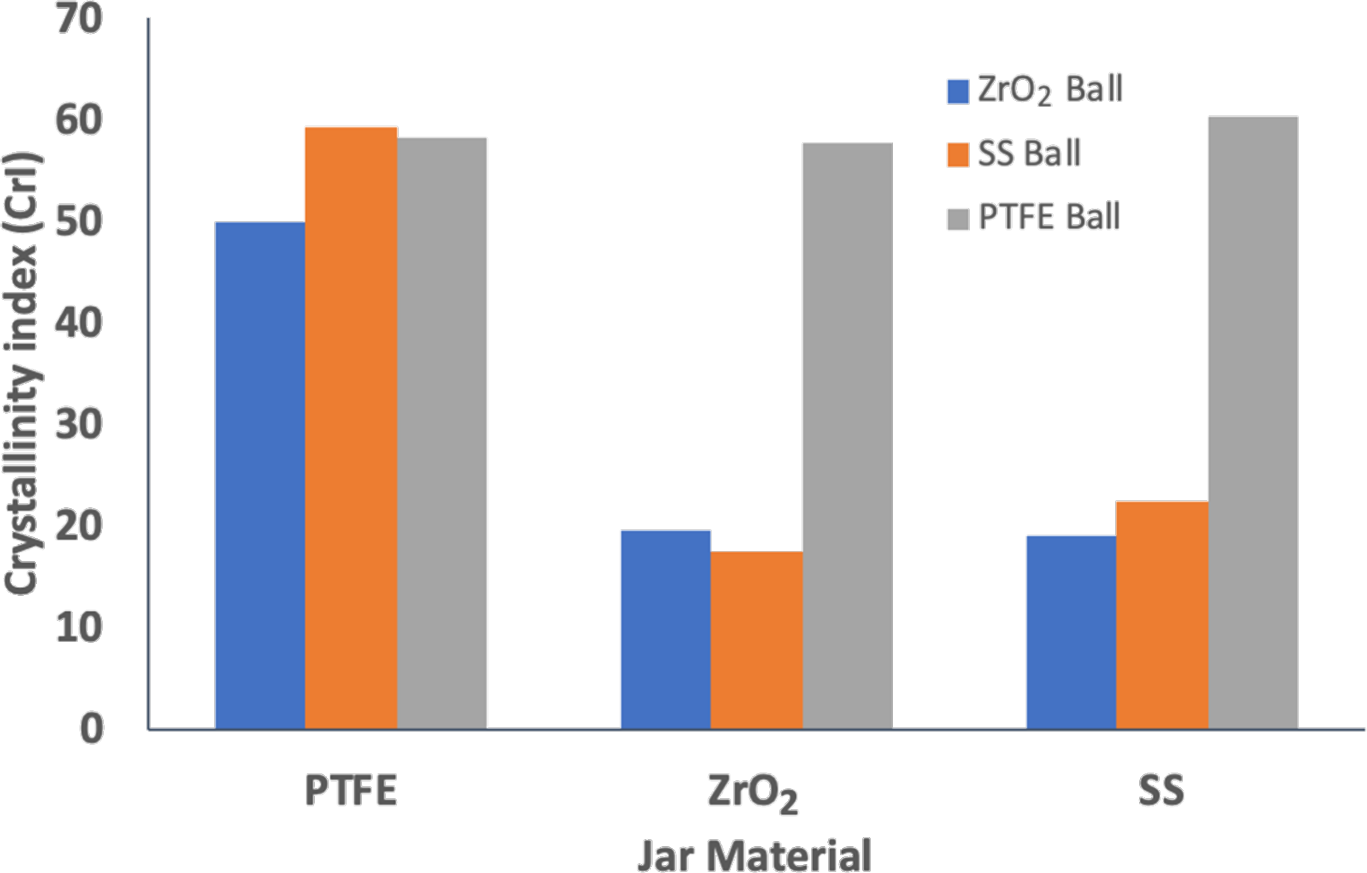
Crystallinity index (%) of chitin milled with 9.5 mm balls of PTFE, ZrO_2_ and SS each used in jars of PTFE, ZrO_2_ or SS. Experimental conditions: 200 mg of chitin, milling time: 30 min, milling frequency 29.5 Hz.

In order to deepen hardness analysis, we decided to vary the ball and jar composition to test materials with hardness below and over the one of chitin cuticle (245 MPa). PTFE has a lower hardness of 30 MPa, while SS and ZrO_2_ have hardness values of 2000 and 10,000 MPa, respectively. Figure 4 displays the crystallinity index of chitin after being milled for 30 min in jars of PTFE, SS or ZrO_2_, fitted with balls of PTFE, SS or ZrO_2_. This study revealed clearly that materials featuring a Vickers hardness higher than the one of the reactive mediums are needed, for both the jar and the ball. If either the jar or the ball is composed of PTFE, the crystallinity of chitin is hardly affected. We thus demonstrated that the milling medium choice is very important in the case of chitin amorphization, and concluded that we have an on/off effect whereby as long as we have a jar and ball of sufficiently hard material, the performance towards amorphization will be essentially the same. Based on this, we wanted to explore the effect of the kinetic energy on the progress of amorphization and thus looked at the effect of ball mass and size.

### Study of chitin amorphization as a function of SS ball size and mass

**Figure 5:**
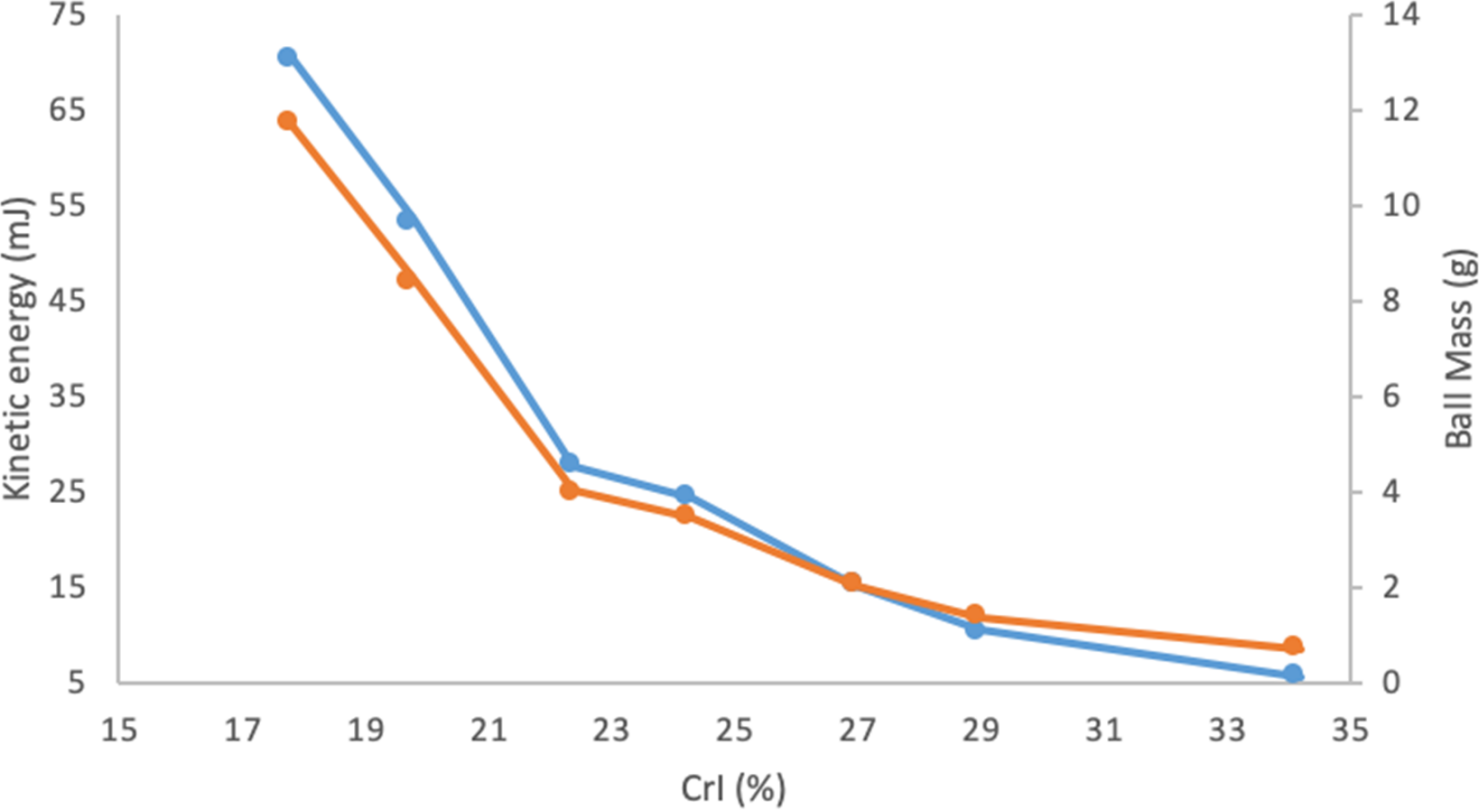
Correlation between the ball mass (orange line) or kinetic energy (blue line) with the crystallinity of chitin after milling in SS jar fitted with 1 SS ball. Experimental conditions: 200 mg of chitin, milling time: 30 min, frequency 29.5 Hz.

To explore the effect of the ball size and mass, we selected one single medium, stainless steel (SS), based on the fact it has sufficient hardness to afford good chitin amorphization. SS is also a common medium used in mechanochemistry, meaning we could easily purchase SS balls of various sizes and masses. Specifically, chitin amorphization was performed with one SS ball in a SS jar, with ball sizes of 7.8, 9.5, 10 and 15 mm. The mass of these balls also differed accordingly. The resulting crystal index (CrI) for chitin after 30 min of milling is provided in Figure 5. This demonstrates that the ball mass has a direct impact on amorphization. In the initial phase, where ball mass is small (1–5 g), crystallinity was drastically reduced from values of 65% to between 23 and 35%. Beyond this point (ball masses of 5 to 12 g), further reduction takes place but was more modest with final values of CrI between 17 and 22%.

This suggested we should further correlate amorphization with kinetic energy formally. To calculate the kinetic energy, we assume a simplified linear motion of the ball in the jar, the time to travel the linear path length based on the frequency of milling, the diameter and mass of the ball. Although not all kinetic energy is transferred from the ball to the powder, the difference is considered negligible [59].

[1] $$E_{k}=\frac{1}{2}mv^{2},$$

where *m* is the mass of the ball, *v* is the average speed of the ball in the jar and *v* is determined by

[2] $$v=\frac{d}{t},$$

where *d* is the overall path length composed of the internal jar path length (varied by jar) less the ball diameter plus the additional distance from movement of the arm swing in the mill (26.4 mm) and *t* is the time it takes to for the mill arm to swing from left to right, considering the frequency used (29.5 Hz) is equal to 1/59 seconds.

We varied the SS ball mass and recorded the performance in terms of chitin amorphization. In Figure 5, we present these results, overlaid with the plotting of the kinetic energy as a function of chitin amorphization. Both the kinetic energy and the mass of the ball are strongly positively correlated with the obtained chitin amorphization. There is a deviation in kinetic energy at higher masses since the ball size decrease the internal milling path length.

### Study of chitin contamination by ball milling medium

**Figure 6:**
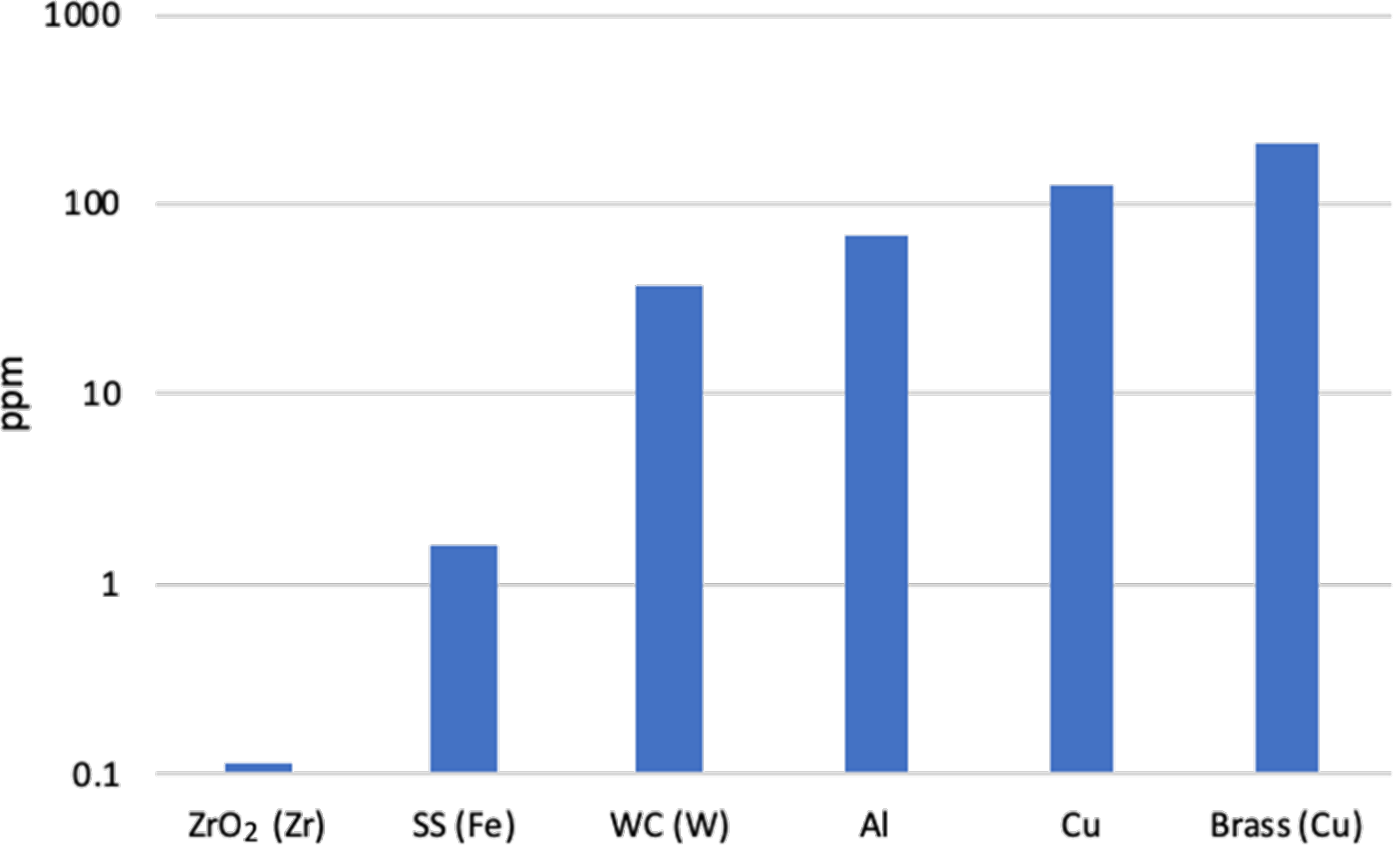
Metal contamination of milled chitin determined by ICP–OES, as a function of the milling medium. The contaminating element is provided in parenthesis.

Chitin is an off-white yellow powder. Milling in ZrO_2_ with a ZrO_2_ ball did not affect its aspect, while milling in SS milling media yielded a gray powder (Figure S4 and Figure S5, Supporting Information File 1). This raises the question of contamination in the milling process, which we explored further. ICP–OES is the technique of choice to establish the metal content in organic matrices. 50 mg of sample was digested in 5 mL aqua regia for 2 h at 90 °C, dissolving the organic biomass and metal contaminants. Milling was conducted in the same manner as amorphization above. The contamination Zr, Fe, W, Al, Cu and Cu was then measured by ICP–OES after milling in ZrO_2_, SS, WC, Al, Cu and brass media (jar and ball), respectively (Figure 6). ZrO_2_ afforded almost no biomass contamination. All other metal containing systems did afford more contamination. SS was actually the least polluting with a contamination value of 1.6 ppm and the others were between 37 and 212 ppm. While Vickers hardness can give a good indication of impact energy transfer from ball to material, it did not correlate as well with the contamination trend. It suggests also that metal release during milling took place from scratching between the ball, the powder and the jar. Contamination by fluorine containing species is harder to establish by ICP–OES, so in order to measure potential release when milling in PTFE, we turned to XPS (Figure S1, Supporting Information File 1). When milling in a PTFE jar with ZrO_2_, fluorine was found in the high concentration of 6.9%. This very high contamination level results from the fact PTFE is very soft and easily scratched by chitin. Clear signs of wear were observed in PTFE jars after several runs, confirming the mechanism.

## Conclusion

Milling is an effective way to amorphize polysaccharides. Amorphization is dependent on factors such as mass, impact area, and material hardness. There is a crossover point for jar and ball hardness, if the jar and ball hardness are below the hardness of the milling mass there is little effect on amorphization, and above this hardness there is a significant degree of amorphization. There is a plateau as well where increased hardness does not increase amorphization as max impact energy transfer to the sample has been achieved. This can give control over mixing or physical modification of the polysaccharides for mechanochemistry experiments and allow for better selection of milling media. Contamination has been demonstrated as potential issue for milling. Milling is considered a neat method, yielding clean products but careful considerations must be taken when selecting milling media for this reason as well.

## Experimental

### Chemicals

Practical grade chitin was purchased from Sigma-Aldrich Co. LLC (St-Louis, MO). Milling media was purchased from McMaster-Carr Supply Co. (Elmhurst, IL) with the exception of ZrO_2_ balls, which were purchased from Glen Mills Inc. (Clifton, NJ). The aluminum and copper balls were carved out of aluminum and copper rods purchased from Home Depot and McMaster-Carr, respectively. The brass jar was carved out of a brass hex rod from McMaster-Carr. The copper jar was made out of copper pipe and copper end caps purchased from Canadian tire. The end caps were domed to provide the standard milling jar internal rounded ends. All other jars were purchased from Retsch.

### Mechanochemical amorphization of polysaccharides

In a typical experiment, 200 mg of polysaccharide, chitin was placed in a jar equipped with one ball and milled in a MM 400 mixer mill for 30 min at 29.5 Hz. The resulting powder was used as is for analysis.

### Powder X-ray diffraction (PXRD)

Sample diffractogram was recorded from 5° to 40° on a zero-background plate using a Bruker D8 ADVANCE X-ray diffractometer equipped using Cu Kα (λ = 1.54 Å) source. Chitin crystallinity was determined by comparing the entire area of the diffractogram (global area) and the area of the crystalline peaks (reduced area). Where CrI (%) = 100%amorphous and %amorphous = [(global area – reduced area)/global area] × 100 [60].

### X-ray photoelectron spectroscopy (XPS)

Samples were analyzed on a Fischer Scientific Kα spectrometer using a spot size of 200 μm, running 5 survey scans at 200 mV for 50 ms residence times, and 10 scans for specific elements, also at residence times of 50 ms. Deconvolution and peak position were determined using Avantage processing software.

### IR

ATR-IR spectra were recorded using a Perkin-Elmer Spectrum 400 for 16 scans from 4000 cm^−1^ to 450 cm^−1^ using approximately 2 mg of sample.

### ICP

ICP analysis was conducted on a Thermo iCap 6500 Duo Series spectrometer. The samples were digested in aqua regia (5 mL) for 2 hours at 90 °C. The samples were then diluted to total volume of 50 mL with DI water and run against standards of the elements of interest.

### Kinetic energy calculation

Kinetic energy of the ball determined by using the mass of the ball and the maximum estimated velocity based on frequency and internal jar milling length plus swing arm movement distance of mixer mill.

## Supporting Information

File 1 Additional experimental data and spectra.
