# Supplementary material for: Mechanochemical amorphization of chitin: impact of apparatus material on performance and contamination
Source: Beilstein J Org Chem. 2019 Jun 5;15:1217–25. doi: 10.3762/bjoc.15.119 (PMC6604705; doi:10.3762/bjoc.15.119)
Supplement: File 1 — Additional experimental data and spectra. [file Beilstein_J_Org_Chem-15-1217-s001.pdf]

BEILSTEIN JOURNAL OF ORGANIC CHEMISTRY

Supporting Information

for

Mechanochemical amorphization of chitin: impact of apparatus material on performance and contamination

Thomas Di Nardo and Audrey Moores

Beilstein J. Org. Chem. **2019,** *15,* 1217–1225. doi:10.3762/bjoc.15.119

Additional experimental data and spectra

License and Terms: This is a supporting information file under the terms of the Creative Commons Attribution License (http://creativecommons.org/licenses/by/4.0). Please note that the reuse, redistribution and reproduction in particular requires that the authors and source are credited.
The license is subject to the *Beilstein Journal of Organic Chemistry* terms and conditions: (https://www.beilstein-journals.org/bjoc)

Table of content

Ball parameters S2

Study of chitin amorphization as a function of SS ball size and mass S2

X-ray photoelectron spectroscopy S3

Powder X-ray diffraction S3

Fourier transform infrared spectroscopy S4

Milled sample picture showing discoloration S5

Ball parameters

Table S1: Vickers Hardness and exact mass of 2 g ball series, standard deviation of 0.237 across all ball masses.

jar type	*Vickers hardness (MPa)*	*total ball mass (g)*
PTFE	30	2.3344
PMMA	170	2.4588
Al	196	1.8319
Cu	369	1.9337
Brass	813	2.2216
SS	2000	2.0595
WC	6080	2.4378
ZrO_2	10,000	2.0112

Study of chitin amorphization as a function of SS ball size and mass

Table S2: Parameters of the balls used in the SS ball size and mass comparison, as well as CrI(%) of chitin milled with them (1 ball, 30 min milling).

Ball Size (diameter, mm)	Ball Mass	Depth @ 30% (mm)	Diameter at depth (mm)	Area of contact at depth (mm^2)	Mass/area (mg/mm^2)	CrI (%)
5.53	0.7055	1.659	5.06	28.76	24.5	34.2
6.97	1.3669	2.091	6.39	45.81	29.8	29
7.8	2.061	2.34	7.15	57.35	35.9	27
9.5	3.464	2.85	8.71	85.1	40.7	24.3
10	4.018	3	9.16	94.17	42.6	22.4
12.64	8.4162	3.792	11.56	150.13	56.1	19.8
14.28	11.71	4.284	13.09	192.23	60.9	17.8

X-ray photoelectron spectroscopy

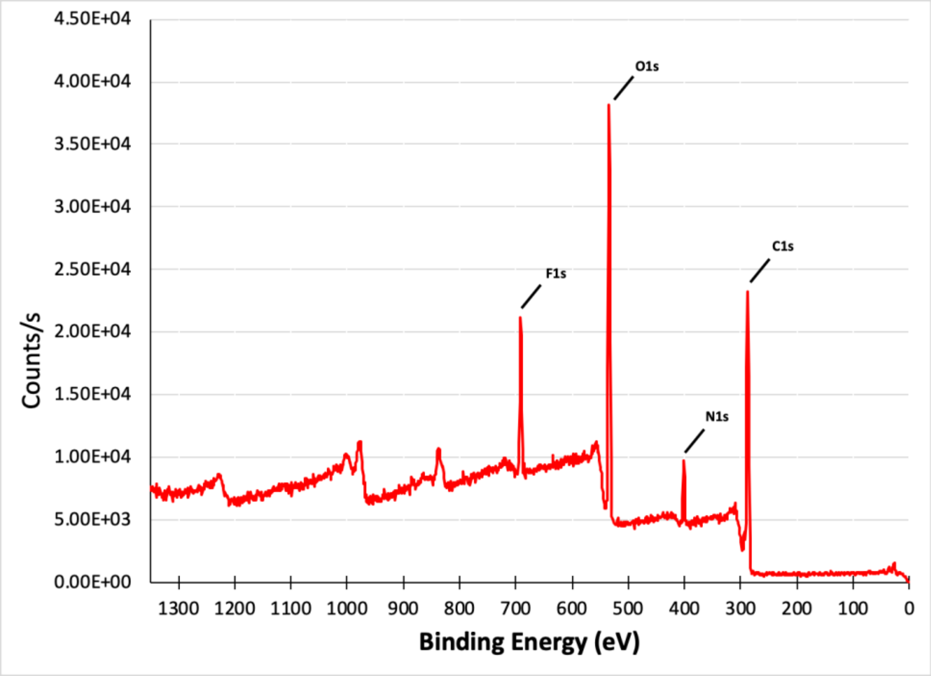

Figure S1: XPS spectrum of chitin milled in PTFE jar with one 10 mm ZrO_2 ball, milling time 30 min.

Powder X-ray diffraction

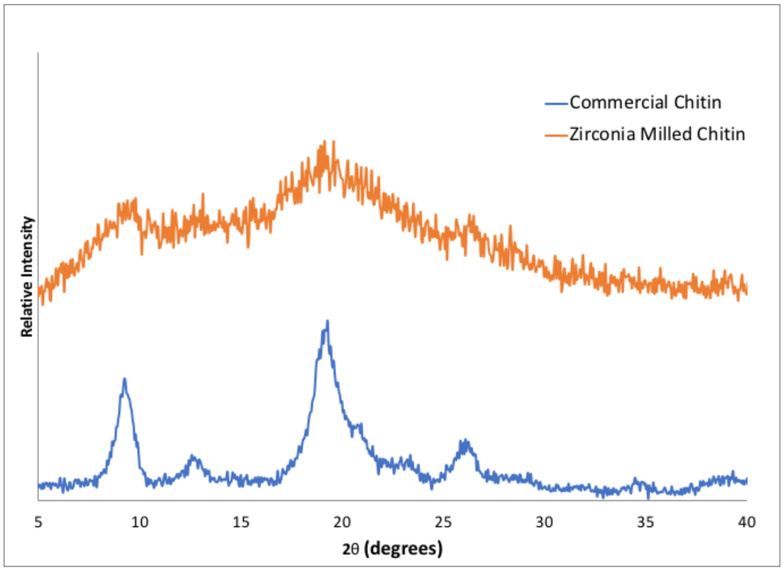

Figure S2: PXRD of biomass, from bottom to top, commercial chitosan (not milled), ZrO_2 milled chitin.

Fourier transform infrared spectroscopy

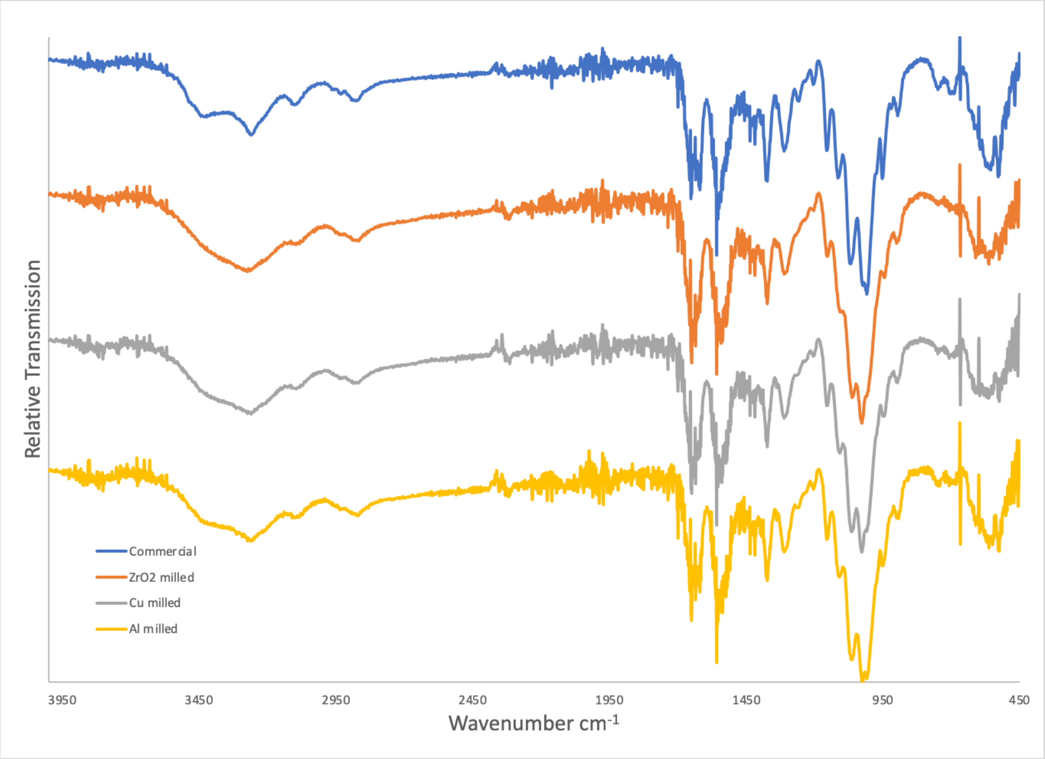

Figure S3: ATR–FTIR of chitin samples commercial chitin, pre and post milling in aluminum, copper, and zirconia.

Milled sample picture showing discoloration

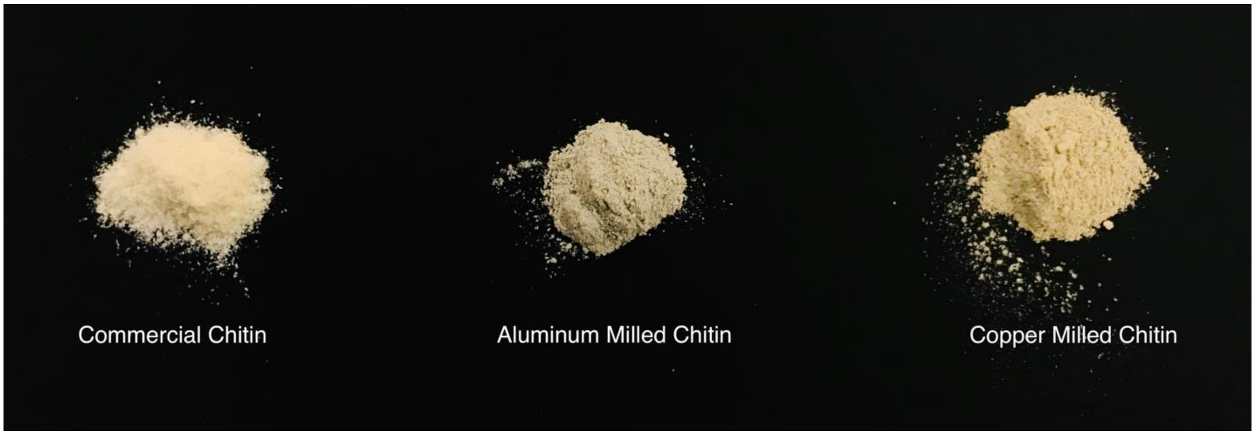

Figure S4: Chitin samples from left to right, commercial chitin (not milled), chitin milled in aluminum jar and ball (30 min, 29.5 Hz), chitin milled in copper jar and ball (30 min, 29.5 Hz).

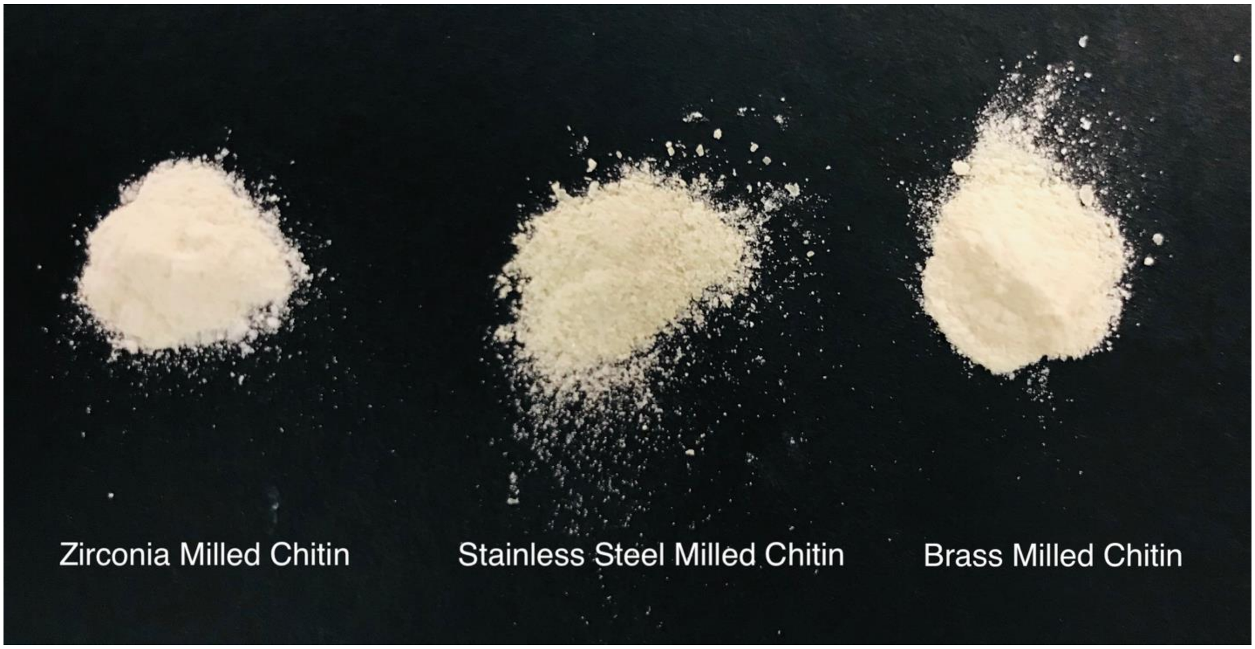

Figure S5: Chitin samples from left to right, chitin milled in zirconia, chitin milled in stainless steel jar and ball (30 min, 29.5 Hz), chitin milled in brass jar and ball (30 min, 29.5 Hz).
